# Vision-based multimodal energy expenditure estimation for aerobic exercise in adults

**DOI:** 10.3389/fphys.2025.1666616

**Published:** 2025-10-08

**Authors:** Lei Jin, Shengxuming Zhang, Mingyao Shi, Long Yu, Mengyao Wang, Mingli Song, Xu Wen

**Affiliations:** ^1^ Department of Sports Science, Zhejiang University, Hangzhou, China; ^2^ College of Computer Science and Technology, Zhejiang University, Hangzhou, China; ^3^ Vivo Mobile Communication Co., Ltd., Dongguan, China

**Keywords:** energy expenditure estimation, computer vision, transformer, skeleton-based action recognition, contactless measurement

## Abstract

**Purpose:**

Estimating energy expenditure (EE) accurately and conveniently has always been a concern in sports science. Inspired by the success of Transformer in computer vision (CV), this paper proposed a Transformer-based method, aiming to promote the contactless and vision-based EE estimation.

**Methods:**

We collected 16,526 video clips from 36 participants performing 6 common aerobic exercises, labeled with continuous calorie readings from COSMED K5. Then we specifically designed a novel approach called the Energy Expenditure Estimation Skeleton Transformer (E3SFormer) for EE estimation, featuring dual Transformer branches for simultaneous action recognition (AR) and EE regression. Comprehensive experiments were conducted to compare the EE estimation performance of our method with existing skeleton-based AR models, the traditional heart rate (HR) formula, and a smartwatch.

**Results:**

With pure skeleton input, our model yielded a 28.81% mean relative error (MRE), surpassing all comparative models. With adopting the heart rate and physical attributes of each participant as multi-modal input, our model achieved a 15.32% MRE, substantially better than other models. In comparison, the smartwatch showed an 18.10% MRE.

**Conclusion:**

Extensive experimentation validates the effectiveness of E3SFormer, aiming to inspire further research in contactless measurement for EE. This study is the first attempt to estimating EE using Transformer, which can promote contactless and multi-modal physiology analysis for aerobic exercise.

## 1 Introduction

Regular physical activity (PA), particularly aerobic exercise with appropriate intensity and frequency, is beneficial to human health (Wang and Xu, 2017). A sedentary lifestyle is associated with an elevated risk of chronic conditions, including obesity, cardiovascular diseases, and diabetes (Kirkham and Davis, 2015; Hamasaki, 2016). Conversely, excessive high-intensity exercise over extended periods may predispose individuals to a higher likelihood of sports-related injuries (Neely, 1998). Energy expenditure (EE), a critical physiological change of exercise, serves as an essential metric for monitoring and regulating daily PA levels and optimizing sports training (Hand et al., 2020). As such, how to estimate EE accurately and conveniently remains a central focus of research in the fields of sport sciences and biomedical.

Traditional methods for estimating EE include the doubly labeled water (DLW) (Westerterp, 2017), indirect calorimetry (IC) (White et al., 2019), and wearable sensors such as heart rate (HR) monitors (Kalkwarf et al., 1989) and accelerometers (Crouter, 2006). The DLW and IC are highly reliable and valid, making them the “gold standard” for EE measurement. However, the DLW only provides total EE over a period rather than activity-specific EE, while IC requires participants to wear a mask connected to a stationary metabolic cart, potentially disrupting movement and performance. Both methods are expensive and limited in practicality (Ainslie et al., 2003; White et al., 2019). HR monitoring, though a mature technology, suffers from reduced accuracy during high- or low-intensity exercise and is susceptible to environmental, emotional, and other external factors (Shcherbina et al., 2017). Accelerometers, widely utilized in PA research due to their convenience and lower cost (Crouter, 2006), are prone to error influenced by sensor placement and movement patterns (Lyden et al., 2011). With the growing popularity of fitness tracking apps, numerous pioneering studies have explored the integration of multiple physiological and biochemical signals from wearable devices (Tikkanen et al., 2014; Villar et al., 2015; Clark et al., 2017). However, the limited availability and discomfort of such devices often restrict their practical application.

In contrast, sports videos can be easily accessed and capture full-body movement. Kinematic parameters such as velocity, acceleration, and joint angles can be extracted from videos to quantitatively describe bodily movement and PA levels, thereby enabling us to estimate EE (Saponaro et al., 2019). Thanks to the advancements in deep learning, many remarkable visual works for action recognition (AR) have emerged (Tran et al., 2014; Simonyan and Zisserman, 2014; Donahue et al., 2015; Carreira and Zisserman, 2017; Tran et al., 2017; Lin et al., 2019; Tran et al., 2019; Feichtenhofer, 2020; Wang et al., 2021; Feichtenhofer et al., 2022; Tong et al., 2022), which has inspired us to estimate EE based on videos. Currently, several studies have already demonstrated the potential of vision-based methods. Tao et al. curated an RGB-Depth video dataset called SPHERE-calorie in a home environment with EE labels obtained from gas exchange measurements, and proposed a method that first performs action recognition and then invokes a specific model based on the identified action category to estimate EE (Tao et al., 2016). Masullo et al. proposed a dual-modal convolutional neural network (CNN) to leverage human silhouette data and accelerometer data to predict EE on SPHERE-calorie dataset (Masullo et al., 2018). Further, a meta-learning method was introduced to achieve personalized EE estimation on the above dataset (Perrett et al., 2022). Nakamura et al. collected an egocentric video dataset complemented by HR and acceleration signals, proposing a multi-modal approach for jointly predicting action category and EE (Nakamura et al., 2017). Peng et al. integrated four widely used AR datasets to acquire Vid2Burn and assigned hourly EE labels through three predefined methods (Peng et al., 2022).

However, current studies in this field exhibit notable shortcomings. First, in the field of AR, existing datasets are constrained by their design, as deep learning models often identify actions based on specific visual contexts within videos rather than focusing on human motion patterns. For precise estimation of EE, a detailed understanding of body movement patterns and their intensity is paramount. Second, existing video datasets for EE estimation either involve activities with limited intensity variation (MET ≤5.0) in controlled environments (household) (Tao et al., 2016; Masullo et al., 2018; Perrett et al., 2022), or their EE labels lack precision and overlook individual differences due to their labels generated from metabolic equivalent (MET) values (Nakamura et al., 2017; Peng et al., 2022).

To construct a comprehensive and authentic benchmark for vision-based EE estimation, recruiting a large number of subjects and collecting video samples of various types of physical activities are indispensable. And the calorimeter based on oxygen consumption ( $\dot{V}O_{2}$ ) is a more ideal manner than MET to measure EE labels. Additionally, the HR and physical attributes of the subject are also correlated with EE.

**FIGURE 1:**
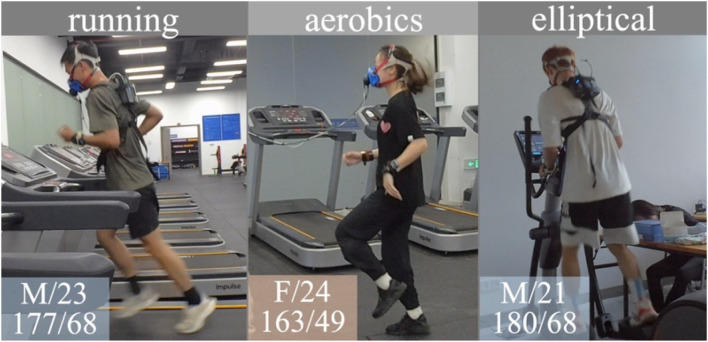
Examples in our dataset. Physical attributes of subjects are at the bottom left corner of each sample, including gender/age, and height (cm)/weight (kg). Abbreviation: M, male; F, female.

Therefore, we introduce an authentic dataset that contains videos of common exercises and corresponding authentic EE labels, with additional information such as HR and subjects’ physical attributes. The EE ground truth labels of our dataset are obtained from the indirect calorimeter COSMED K5. The dataset is further enriched with multi-modal data, including real-time HR and physical attributes of participants. Some examples from the dataset are illustrated in Figure 1.

Based on this dataset, we propose a novel method for estimating EE based on human skeleton and Transformer architecture, termed as *E3SFormer*, which is an abbreviation for Energy Expenditure Estimation Skeleton Transformer. First, we utilize an off-the-shelf pose estimation method to extract the skeleton sequence of the exerciser from videos (Cai et al., 2020). Then, we input this skeleton sequence into a Spatio-Temporal Fusion Transformer backbone to extract features. The extracted features, which encapsulate information across both the temporal dimension and the spatial dimension (*i.e.*, human joint dimension), are subsequently fed into two distinct Transformer network branches. One branch is dedicated to predicting the action category, while the other focuses on estimating EE. Different from the prior video-based or skeleton-based methods that strongly rely on action recognition accuracy, we introduce the independent EE regression branch into the Transformer architecture for the first time. This dual-branch architecture ensures a comprehensive integration of motion dynamics and physiological context, enabling accurate and robust EE estimation.

Intuitively, we believe that the movement features of certain specific joints on the human body are key to action classification, and the intensity or temporal dynamics of these joints’ motion have a stronger correlation with EE. For instance, regardless of hand movements, the action of running requires a rapid alternation of stepping forward and backward with both legs. Therefore, we transfer the attention of each joint from the AR branch to the EE regression branch to enhance its performance. Based on the fact that different individuals will have varying EE when engaging in the same type and intensity of exercise, using only video clips or skeleton sequences to accurately predict EE is inadequate. More personalized data are required for this purpose. Therefore, in the network design, we added a multi-modal data input module to achieve more personalized EE estimation with subjects’ real-time HR and physical attributes.

Thus, focusing on aerobic exercise, this study aims to establish an authentic vision-EE benchmark and design a neural network supporting multi-modal data input for the EE estimation task. Furthermore, we conduct experiments to demonstrate the superiority of our method, aiming to inspire further research in vision-based, contactless, and intelligent EE estimation.

## 2 Methods

### 2.1 Participants and data collection

Thirty-six healthy participants were recruited for the study. Ethics approval was obtained before the commencement of this research. Each participant signed a *Written Informed Consent* and a *Sports Health Survey Privacy Policy* agreeing to share their data for research purposes. The participants were also asked to complete the *Exercise Risk Screening Questionnaire* prior to their participation in this study; those with any contraindications to exercise were excluded from the study. Six popular types of indoor exercise in daily life are included: *running*, *riding*, *elliptical*, *skipping*, *aerobics*, and high-intensity interval training (*HIIT*). For diversity, the first four types are further set three speed levels (slow, medium and fast), subdividing the dataset into 14 activity classes. *Running* is testing on a treadmill with a 0% incline and speed settings of 8, 10, and 12 km/h for males and 7, 9, and 11 km/h for females. The resistance for *riding* is set to 1 gear (approximately 8 kg), maintaining speeds around 60, 80, or 100 RPMs (revolutions per minute). The speed of the *elliptical* is set to 30, 50, or 70 RPMs, and the resistance is adjusted to the maximum level each participant could sustain at the corresponding speed. *Skipping* was performed at three speeds: 60, 100, and 140 RPMs. The instructional videos of *aerobics* and *HIIT* were downloaded from the internet for the participants to follow and practice. For each test, the participant was required to exercise at a constant speed for 30 min. Before and after the exercise, they were instructed to sit quietly for 5 min each to record rest and recovery data. Throughout this procedure, the participants were required to wear the COSMED K5 portable metabolic system (K5 for short), the Polar H10 heart rate band, and a smartwatch for continuous monitoring.

The K5 measures respiratory gas exchange using the dynamic mixing chamber (DMC) or breath-by-breath (B × B) technique and then calculates EE based on IC, which is the most effective and accurate approach for estimating EE during rest and aerobic exercise (Crouter et al., 2019; White et al., 2019). The Polar H10, a chest-worn HR monitor, was synchronized with the K5. Both ground truth sources are recognized as the “gold standard” and have been widely used in sports research. Simultaneously, all RGB videos were captured by the EZVIZ S2 camera at 2.7k raw resolution and 30 fps. Totally, over 112 original sports videos were collected from multiple viewpoints.

### 2.2 Data preprocessing and split

Given the high resolution of the original videos, we down sampled them to an 856 × 480 resolution for facilitating processing. The samples have two kinds of EE measurement ways (DMC and B × B); therefore, for the sake of uniformity and ease of processing, the videos were cut into clips every 10 s and then labeled with EE and HR. For the DMC video samples, the original records are sufficient to assign the clip labels; for the B × B samples, the EE and HR records were averaged over 10-s intervals as the labels of the video clips. In this manner, we obtained 17,260 video clips annotated with EE and HR labels, and matched them with the physical attributes of the participants.

**FIGURE 2:**
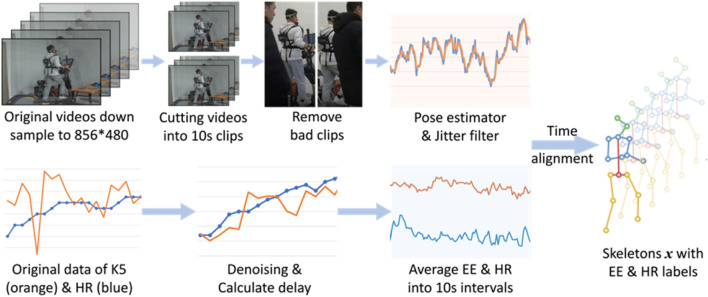
The diagram of data preprocessing.

Considering that there is a temporal delay between the actual occurrence of EE in the muscles and its recording by the metabolic system (Hughson et al., 2001), we calculated a mean delay time for each participant similar to (Blake and Wakeling, 2013) and revised the EE labels before cutting the videos. Then, we extract the human body skeleton sequence using the combination of the RTMPose (Cai et al., 2020) and the RTMDet models (Lyu et al., 2022). We also write a script to filter out the unrelated moving individuals automatically. A 10-s video clip contains 300 frames, and captures at least 2 repetitions of movements, which contain complete exercise cycles. After manually removing a small number of bad video clips, we finally obtained 16,526 video clips as our dataset. Finally, we chose to use the Euro filter (Casiez and And Vogel, 2012) to filter the obtained skeleton data so that reduce the jitter. The preprocessing steps above are shown in Figure 2.

The application of EE estimation based on video requires the model to have a strong generalization on individuals not seen in the training set. In order to evaluate the generalization of the model, we divided the dataset into training, validation, and test sets according to the participants. Specifically, we randomly divide the 36 participants in a roughly 6:2:2 ratio, assigning 22 participants to the training set, with 7 participants each in the validation and test sets. Accordingly, the number of video clips in the training, validation, and test sets are 10,049 and 3,234 and 3,243, respectively. This cross-subject data split ensures that the participants used for evaluating the model’s performance are not seen during the model training process, which allows for an effective assessment of the model’s generalization ability.

### 2.3 E3SFormer: energy expenditure estimation skeleton transformer

Accurately estimating EE requires fine-grained analysis of video, which is a computationally intensive task. Traditional video understanding methods generally sample a small number of frames in each video (Tran et al., 2014; Simonyan and Zisserman, 2014; Carreira and Zisserman, 2017; Feichtenhofer et al., 2018), which is inadequate for predicting precise EE of human motion. If all frames of a video clip are inputted into these methods, the GPU memory usage and inference time will be excessive, making it unfavorable for practical applications. Furthermore, irrelevant stuff and background in the video may affect the prediction of EE. Therefore, we use the human body skeleton sequence of subjects extracted from video clips when data preprocessing as input, and then adopt a human skeleton-based method to accurately estimate EE on our dataset and reduce computational cost and inference time.

**FIGURE 3:**
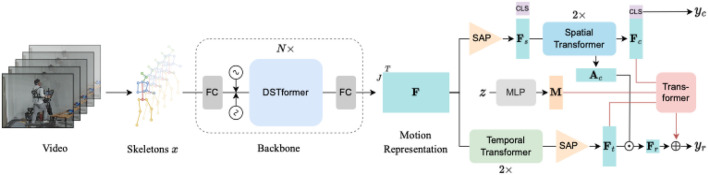
Framework of E3SFormer. The human skeleton sequence *x* is extracted using a pose estimator from the video and then fed into a backbone to obtain motion representation F . It is then sent to an action recognition branch (upper) and an EE estimation regression branch (lower). The category-related joint-specific attention $A_{c}$ from the action branch is transferred to the EE estimation regression branch to boost its performance. The multi-modal data *z* is used for more personalized EE estimation.

The overall procedure of our model E3SFormer is illustrated in Figure 3, including a backbone and two branches for action recognition and EE regression, respectively. The entire network is based on the Transformer architecture (Zhu et al., 2023). The backbone uses a spatial-temporal fusion for extracting spatial and temporal features of an inputted human skeleton sequence. Afterward, the features are fed into the two different branches for different tasks simultaneously. The attention of each joint in the action recognition branch, termed as $A_{c}$ , is transferred to the EE regression branch to facilitate precise EE regression.

#### 2.3.1 Spatio-temporal motion feature extraction

The key component of the backbone is a Dual-stream Spatio-temporal Transformer (DSTformer) block. One DSTformer block consists of two different branches. The first branch initially performs a Transformer along the spatial (joint) dimension, followed by a Transformer on the temporal dimension. The second branch switches the order of these two Transformers. The result of these two branches is fused through adaptive weights produced by an attention regressor. Each branch of DSTformer has the capability of modeling comprehensive spatio-temporal information, and different branches are interested in different spatio-temporal aspects. The fusion operation can dynamically balance the results of these two branches.

Specifically, we define the input skeleton sequence as $x\in R^{T\times J\times C_{in}}$ , where *T* is the temporal sequence length, *J* is the number of body joints, and *C*_*in*_ is the channel number of input. Specifically, *C*_*in*_ = 3 in here, the first and second channels are the x-coordinate and y-coordinate of body joints respectively, and the third channel is the visibility confidence of each joint offered by the pose estimation method (Cai et al., 2020). The skeleton sequence *x* is projected to a high-dimensional feature $F^{0}\in R^{T\times J\times C}$ , and concatenated with a pretrained spatial position encoding $P_{S}\in R^{1\times J\times C}$ and a temporal position encoding $P_{T}\in R^{T\times 1\times C}$ . Then the input feature is fed into the backbone that contains *N* DSTformer blocks to get the motion representation $F\in R^{T\times J\times C}$ . *C* denotes the channel of features used in the backbone and thereafter branches. The obtained motion representation F is then fed into two transformer branches for both action recognition and energy expenditure regression.

#### 2.3.2 Spatial-based action recognition

For the action recognition branch, we first use a Self-Attention Pooling (SAP) layer to squeeze the temporal dimension *T* of F , which is defined as Equation 1:

$$\text{SAP}\left(F_{j}\right)=\sum_{t=1}^{T}\frac{\exp\left(\text{FC}\left(F_{j}^{t}\right)\right)}{\sum_{t^{\prime }=1}^{T}\exp\left(\text{FC}\left(F_{j}^{t^{\prime }}\right)\right)}\cdot F_{j}^{t},$$ (1)

where $F_{j}$ is the slice of F along the joint dimension, FC is a Fully Connected layer. The result of this SAP layer is denoted as $F_{S}\in R_{J\times C}$ , which is concatenated with a class token (CLS) and fed into a two Spatial Transformer (ST) layer to model the relation shape among the joints. The ST aims to perform Transformer operation along the joint dimension, the key component of which is the Multi-Head Self-Attention (MHSA). MHSA is calculated in a similar way as in MotionBERT (Zhu et al., 2023). Residual connection is used to the MHSA result, which is fed into a multilayer perceptron (MLP), and followed by a residual connection. The Pre-LayerNorm trick is used for both MHSA and MLP.

#### 2.3.3 Joint-specific attention for enhanced energy expenditure regression

Every token in the action recognition branch leverages its query to calculate the similarity of all keys to form the attention matrix, representing which tokens should be concerned. The CLS token is used to classify action, so in our intuition, which joints are important for a certain action category can be represented by the attention of the CLS token. Therefore, the average of multi-head attention of CLS token in the second ST, which if termed as category-related joint-specific attention $A_{c}\in R^{J}$ , is used to signify the importance.

For the EE prediction branch, there are two Temporal Transformer (TT) layers followed by an SAP layer. The only difference between ST and TT is that TT is performed along the temporal dimension of each joint. The result can be denoted as $F_{t}\in R^{J\times C}$ . To gain the enhanced representation for regression, we use $A_{c}$ as a weight to calculate a weighted sum of $F_{t}$ along the joint dimension, resulting in $F_{r}\in R^{C}$ . For the integration of multi-modal data z including heart rate and physical attributes, an MLP is used to extract feature M of them. Then, it is concatenated with the $F_{t}$ and the result of action recognition branch $F_{c}\in R^{J\times C}$ without CLS token and fed into a Transformer layer. The result as well as $F_{r}$ is used to regress EE.

We use the Cross-Entropy Loss $L_{c}$ to train the action recognition branch, together with L1 Loss $L_{r}$ to train the EE regression branch. The overall loss function is as Equation 2:

$$L=L_{r}+\alpha L_{c},$$ (2)

where α is a hyperparameter. To balance the action recognition loss and EE regression loss, we set α=0.2 for our E3SFormer framework.

### 2.4 Experiment setup

#### 2.4.1 Comparison methods

We compared the proposed E3SFormer with two skeleton-based action recognition frameworks, namely, ST-GCN (Yan et al., 2018) and PoseConv3D (Duan et al., 2021), on our dataset. Among them, the PoseConv3D is based on convolutional neural networks (CNN), while the ST-GCN is based on graph convolutional networks (GCN). We modified the output channel of the last Linear layer originally for classification to 1 for EE regression. Besides, we altered the input channel of PoseConv3D from *J* to 3 (*J* is the number of body joints), and used the sequence of RGB video frames as input to simply compare the performance of the skeleton-based and video-based approach. The altered framework is designated as RGBConv3D. When using multi-modal data as input, the smartwatch prediction results were added for comparison.

#### 2.4.2 Training details

We used the pretrained weight of MotionBERT (Zhu et al., 2023) to initialize the backbone. However, the length of pretrained temporal position encoding $P_{T}$ is insufficient for our fine-grained task that has a quite long skeleton sequence. Therefore, we performed linear interpolation on the T dimension of $P_{T}$ from the original number to a longer number to accommodate longer input sequences. Our model and comparison models were implemented by PyTorch and optimized by Lion optimizer (Chen et al., 2024) with a learning rate of 10^−4^, weight decay of 5 × 10^−4^, and cosine annealing as the learning rate decay schedule. We trained all the settings for 50 epochs with a batch size of 16, except the two CNN-based models, PoseConv3D (Duan et al., 2021) and RGBConv3D. Considering the larger GPU memory usage of these two models, we set the batch size of these two models to 8. For all the skeleton-based models, the joint coordinates were normalized to the range of [-1,1]. The random horizontal flipping was applied as the data augmentation. The experiments were running on two Intel Xeon 4215R CPUs with 128G memory and one NVIDIA RTX A6000 GPU.

#### 2.4.3 Evaluation metrics

We adopted L1 Loss to train every model for EE regression, which is also known as Mean Absolute Error (MAE). In addition to MAE, Mean Relative Error (MRE), Pearson Correlation Coefficient (PCC), and Coefficient of Determination ( $R^{2}$ ) were also used as evaluation metrics for the model.

## 3 Results

### 3.1 Participants demographics

Table 1 summarizes the physical attributes of the participants. A total of 36 healthy adults gave consent to participate, including 15 males and 21 females, with an average age of 23.3 years, mostly recruited from the university.

**TABLE 1:** The physical attributes of participants.

Characteristics	Total sample
Gender (m/f)	15/21
Age (yr)	23.3 ± 3.0
Hight (cm)	172.2 ± 8.7
Weight (kg)	65.8 ± 14.9
BMI (kg/m^2^)	22.0 ± 3.4

Data presented as mean ± SD.SD, standard deviation; BMI, body mass index.

### 3.2 Dataset statistics

**FIGURE 4:**
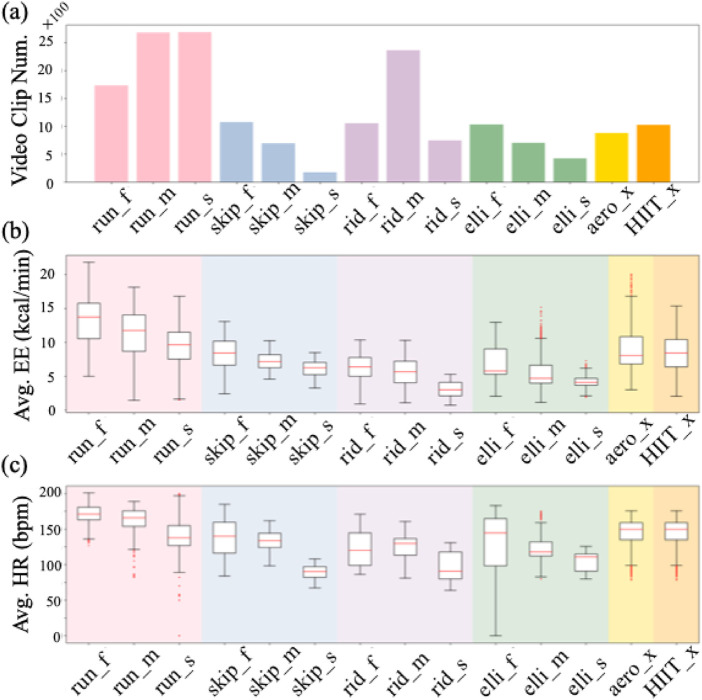
Statistics of our dataset. **(a)** The number of video clips. **(b)** Average energy expenditure (EE) for each class. **(c)** Average heart rate (HR) for each class. The _f, _m, and _s denote fast, medium, and slow speed respectively, while _x denotes no speed label.

The dataset contains a total of 6 major categories of aerobic exercises, which are further divided into 14 classes based on speed. Figure 4a illustrates the distribution of video clips across each class. *Running* is the most frequent category, comprising a total of 7096 clips, with 1733 in fast (_f), 2676 in medium (_m), and 2687 in slow (_s) speed variations. The average number of video clips per class is 1232.9. Figures 4b,c depicts the distribution of EE and HR measurements across each exercise class, respectively. Generally speaking, the higher the exercise intensity, the higher the EE and HR. Among the categories, *running_f* shows the highest average EE and HR, while *riding_s* has the lowest average EE and HR.

### 3.3 Joint-specific attention

**FIGURE 5:**
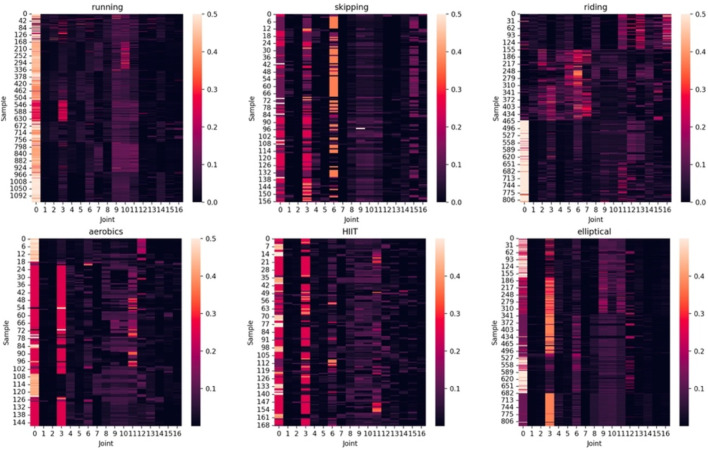
Category-related joint-specific attention $A_{c}$ for each example of each general exercise category in the test set.

Figure 5 shows the heatmaps of category-related joint-specific attention $A_{c}$ for each general exercise category in our test set. The six subplots correspond to six categories of exercise, titled above each subplot. The x-axis of each subplot represents joint indexes, and their specific correspondence with human body joints, which is originated from the Human3.6M dataset (Ionescu et al., 2014). The y-axis represents sample indexes; thus, each row of the subplots represents an $A_{c}$ of a specific sample. The brighter the heatmap, the larger the $A_{c}$ value, indicating that the joint is more important for its exercise category.

### 3.4 Pure skeleton results

As shown in Table 2, when we only leverage the human skeleton sequence as input, the proposed E3SFormer surpasses all comparison methods on most evaluation metrics, except for $R^{2}$ . But the $R^{2}$ still ranks second among all the methods and is close to the first (0.5118 compared to 0.5175). These results demonstrate the effectiveness of our method.

**TABLE 2:** EE regression results with pure skeleton sequence as input.

Method\Metric	MAE ↓	MRE (%) ↓	PCC ↑	$R^{2}↑$
ST-GCN	2.1939	36.42	0.6632	0.3722
PoseConv3D	2.0670	33.03	0.7232	**0.5175**
RGBConv3D	2.5408	42.93	0.5186	0.2663
**E3SFormer**	**2.0304**	**28.81**	**0.7528**	0.5118

The ↓ indicates the lower the better, and the ↑ indicates the higher the better. MAE, mean absolute error; MRE, mean relative error; PCC, Pearson correlation coefficient; $R^{2}$ , coefficient of determination. The values in bold indicate the best results.

### 3.5 Multi-modal input results

Based on the fact that different individuals will have varying EE values when engaging in the same type and intensity of exercise, using only video clips or skeleton sequences to accurately predict EE is inadequate. More personalized data are required for this purpose.

For all the comparison methods, we leverage a three-layer multi-layer perceptron (MLP) to extract a feature of HR and anthropometric characteristics of each input sample. The hidden layers and output layer of the MLP have the same number of channels as the output channels of each backbone in these methods. The extracted attribute feature is concatenated with the backbone feature and fed into a fully connected layer to predict EE. When augmented with HR and anthropometric characteristics, the model performances are much better than without these multi-modal data, shown in Table 3.

**TABLE 3:** EE regression results with multi-modal data as input.

Method\Metric	MAE ↓	MRE (%) ↓	PCC ↑	$R^{2}↑$
ST-GCN	1.4895	23.06	0.8637	0.7169
PoseConv3D	1.3939	21.52	0.8976	0.7861
RGBConv3D	1.7382	28.83	0.8988	0.7048
smartwatch	1.4582	20.79	0.8271	0.6763
**E3SFormer**	**1.1039**	**15.32**	**0.9082**	**0.8225**

The ↓ indicates the lower the better, and the ↑ indicates the higher the better. MAE, mean absolute error; MRE, mean relative error; PCC, Pearson correlation coefficient; $R^{2}$ , coefficient of determination. The values in bold indicate the best results.

In addition, as the most popular wearable devices, smartwatches also use multi-modal data for EE prediction. For comparison, the subjects were asked to wear a smartwatch during dataset collection. We calculated the evaluation metrics of the smartwatch on the test set and added the results in Table 3.

### 3.6 Ablation study

Table 4 shows the ablation study that we conducted. The upper half of the table is experiments using only heart rate (HR) and physical attributes (Attr) to predict EE. The formula is given by the *American College of Sports Medicine* to estimate EE based on these data (Medicine, 2013). The parameters of the formula differ for males and females. For males, the formula is as follows:

$$\text{EE}=\frac{\left(0.6309\times HR+0.1988\times W+0.2017\times A-55.0969\right)}{4.184},$$

while for females, the formula is:

$$\text{EE}=\frac{\left(0.4472\times HR+0.1263\times W+0.074\times A-20.4022\right)}{4.184},$$

where EE denotes the energy expenditure (*kcal/min*), *HR*, *W*, and *A* denote heart rate, weight, and age, respectively. The rest three rows are the experiments using a three-layer MLP to predict EE according to the specified data. The channel number of the hidden layers is 512. The lower half of Table 4 is the ablation study of E3SFormer’s action recognition branch with the category-related joint-specific attention. The “w/o AR” refers to replacing the joint-specific attention with average pooling for averaging regression outputs.

**TABLE 4:** Ablation study of our method.

Ablation\Metric	MAE ↓	MRE (%) ↓	PCC ↑	$R^{2}↑$
Formula	3.5047	65.02	0.7767	−0.1493
Only HR	2.7904	32.75	0.7871	0.0268
Only Attr	3.0140	58.41	0.5812	0.0416
HR + Attr	1.5276	25.78	0.8712	0.7297
w/o MM w/o AR	2.1071	39.22	0.7155	0.4704
w/o MM w/AR	2.0304	28.81	0.7528	0.5118
w/MM w/o AR	1.8705	29.88	0.7260	0.5035
w/MM w/AR	**1.1039**	**15.32**	**0.9082**	**0.8225**

“Formula” denotes using a predefined set of formulas to calculate EE based on heart rate and physical attributes. The w/o MM and w/ MM denote without and with heart rate and physical attributes as multi-modal data, respectively. The w/o AR and w/ AR denote without and with action recognition branch, respectively. The ↓ indicates the lower the better, and the ↑ indicates the higher the better. MAE, mean absolute error; MRE, mean relative error; PCC, Pearson correlation coefficient; $R^{2}$ , coefficient of determination. The values in bold indicate the best results.

### 3.7 Multi-view analysis

In order to analyze our model’s sensitivity to the viewpoint, we divided the test set into subsets based on the viewpoint and tested the model performance on each subset separately. Due to the use of flip data augmentation during training, there is effectively no difference between left and right viewpoints. Therefore, the left and right perspectives were combined into a single subset. The sample numbers of front, back, left & right viewpoints are 157, 1803, and 1283, respectively, and the results are shown in Table 5.

**TABLE 5:** Our model metrics of different viewpoints.

Viewpoint\Metric	MAE ↓	MRE (%) ↓	PCC ↑	$R^{2}↑$
front	1.3577	17.95	0.7557	0.5191
back	1.3089	17.50	0.9031	0.7723
left & right	1.1516	18.73	0.9530	0.8615

The ↓ indicates the lower the better, and the ↑ indicates the higher the better. MAE, mean absolute error; MRE, mean relative error; PCC, Pearson correlation coefficient; $R^{2}$ , coefficient of determination.

## 4 Discussion

### 4.1 Visualization of joint-specific attention

All subplots of Figure 5 reveal that in most samples, the hip (joint 0) has a relatively high $A_{c}$ value, except for half of the samples in riding. As the joint closest to the body’s center of gravity, the hip naturally holds significant importance because it can represent the overall movement of the body. For instance, the frequency of vertical oscillation and the speed of horizontal movement of the body’s center of gravity are important indicators for accessing the EE and running economy of a runner (Saunders PU et al., 2004; Barnes and Kilding, 2015). The reason why half of the samples in *riding* do not show this pattern, in our opinion, is that the hips are seated on the seat of the stationary bike, thereby remaining motionless. Actually, we also drew the heatmaps of the validation set, and found that the hips of most of the riding examples in the validation set are not important.

Apart from the hip, different exercises show different patterns of $A_{c}$ , and most samples within the same exercise category exhibit similar patterns. The left foot (joint 3) and right foot (joint 6) are crucial for *skipping* because this kind of exercise primarily relies on the force generated by the calf muscles. For a subset of samples in *riding*, all joints of the lower limbs (from joint 1–6) show relatively high importance, as it requires exerting force with the thighs to pedal. In *running*, *aerobics*, and *HIIT*, the joints of the upper limbs also hold a certain level of importance because these exercises are accompanied by movements of the upper limbs.

### 4.2 Regression results of energy expenditure

As shown in Table 2, the PoseConv3D (Duan et al., 2021) ranks first on $R^{2}$ and performs relatively better on other evaluation metrics compared to ST-GCN (Yan et al., 2018), exhibiting the superior capability to extract fine-grained features in our task. We conjecture that this is because the issue of over-smoothing in GCNs results in a diminished ability to extract fine-grained features in the deeper layers of the network. Accurate estimation of EE, however, requires precise capture of the displacement of each joint to measure muscle contractions, a capability where CNN excel.

Despite being a CNN, the RGBConv3D performs much worse compared to ST-GCN and PoseConv3D. The main reason, in our opinion, is that the inputs of RGBConv3D are RGB video clips that contain irrelevant objects, other people, and various backgrounds, which may disturb the prediction of EE. By contrast, PoseConv3D renders the joint coordinates to the video space as the input of CNN, focusing on human body movement while disregarding the influence of background factors.

With the help of multi-modal data, the performances of all methods improved significantly, as shown in Table 3. E3SFormer ranks first on all of the evaluation metrics, owing to a meticulously designed architecture. The gap between CNN-based and GCN-based methods becomes less pronounced. The PoseConv3D does not stand out on the evaluation metrics representing prediction accuracy (MRE and MAE), but performs well on the evaluation metrics related to correlation (PCC and $R^{2}$ ). The PCC of RGBConv3D is quite high while the $R^{2}$ is relatively lower, which is related to the worst performance on MRE and MAE, showing a high correlation but low prediction accuracy. The incorporation of multi-modal data boosts the prediction accuracy of all methods. However, according to the two analyses above, due to the structural advantages of CNN, CNN-based methods exhibit better predictive correlation.

Table 3 also shows that all the metrics of our methods surpass those of the smartwatch. We conjecture that it is because the smartwatch is less sensitive in the early stage of exercise. The smartwatch failed to sense the exercise intensity and estimated EE as 0 due to the slow increase in the HR and EE values of the subject (approximately 0.5∼2.0 kcal/min). This failure is also attributed to the dominance of the lower limbs in most of the testing exercises (such as *running*, *riding*, and *elliptical*) and the small movement amplitude of the wrist, resulting in low prediction values for the smartwatch. The above results demonstrate the suitability of the proposed model for product integration.

As for the ablation study results, the upper half of Table 4 shows that the neural networks are more appropriate than the predefined formula for this task. By using nonlinear activation functions, MLP is able to learn and model nonlinear relationships and complex functions, which makes it capable of dealing with nonlinear problems. Besides, both using only heart rate and using only physical attributes are not sufficient to produce an acceptable result, indicating that EE is related to a combination of both, rather than either one alone.

The lower half of Table 4 show that without the joint-specific attention, the performance will degenerate substantially. After using the category-related joint-specific attention from the action recognition branch, the MRE will be reduced more than 10%, demonstrating the importance of it. It also proves that the motion features of certain specific joints on the human body over time have a great correlation with EE estimation. From the sports videos, we can capture these category-related joint-specific attention. From the sports videos, we can capture the motion characteristics of category-related key joints through the attention mechanism, so as to predict the motion intensity and calculate EE in a more comprehensive and precise way.

In Table 5, it can be seen that the MRE differences across various viewpoints are small. The other three metrics of left & right superior, possibly because the side perspective provides more information on limb movements. The PCC and $R^{2}$ of the front viewpoint are low due to the limited sample size, and most are *aerobics* and *HIIT* videos, which are highly complicated and difficult to predict EE. Therefore, the model is indeed slightly sensitive to the viewpoint, but the results are also influenced by other factors.

### 4.3 Limitations and future works

This research is an effective attempt and application of artificial intelligence (AI) in EE estimation field, but it indeed has limitations. First, the age distribution of participants is not wide enough. Thus, more children and elderly volunteers should be recruited to expand our dataset. Second, the data were all collected in the gym scenario, potentially limiting the practicability of the E3SFormer in outdoor settings. The research in the vision-based EE estimation field is in its early stage. This paper focuses on data collection in indoor settings to minimize the interference of external factors, such as wind speed, ground slope, temperature, etc. In the future, additional outdoor exercise video data will be collected to enhance the universality and robustness of our model. Third, the real-time performance of the E3SFormer still needs improvement. Since the input of the E3SFormer is a skeleton sequence rather than original videos, we need to preprocess the video clips and use the pose estimator to extract human skeletons. Currently, under our experimental conditions, the inference speed of the E3SFormer is only 0.08∼0.20 s for 300-frame clips, but the preprocessing and pose estimator take 4.3∼6.0 s, which has not been optimized for deployment. Future researchers can optimize inference speed by model quantization, model pruning, knowledge distillation, and designing more efficient model architectures, which will improve its practicability for product integration. If a more efficient architecture is designed in the future, the model will be further optimized and deployed, possibly integrated into a product that can be applied to contactless fitness training monitoring and even predicting patients’ physical activity levels without interference in clinical practice.

## 5 Conclusion

This work is the first contribution to estimating energy expenditure using Transformer architecture. We first curate an authentic benchmark including 16,526 aerobic exercise videos, labeled with the COSMED K5 calorimeter, the heart rate and physical attributes of each subject. Based on this dataset, we proposed a dual-branch network E3SFormer that utilizes human skeleton data from videos to regress energy expenditure. The attention of each joint in the action recognition branch is transferred to the energy expenditure regression branch to facilitate precise regression. Comprehensive experiments exhibited the effectiveness of the E3SFormer, aiming to inspire further research in contactless and vision-based energy expenditure estimation. The outstanding results achieved by the use of multi-modal data further demonstrate the signification application of AI multi-modal models in contactless motion analysis.

## Data Availability

The raw data supporting the conclusions of this article will be made available by the authors, without undue reservation.
